# Clinical, laboratory and genetic factors associated with smoking in a Brazilian Sickle Cell Disease (SCD) cohort

**DOI:** 10.1371/journal.pone.0332305

**Published:** 2025-09-26

**Authors:** Theomario Cruz, Beatriz Araujo Oliveira, Mina Cintho Ozahata, Isabel Gomes, André Belisário, Anna Bárbara Carneiro-Proietti, Paula Loureiro, Sheila de Oliveira Garcia Mateos, Cecília Salete Alencar, Rodrigo Oliveira, Frederico L. A. Fernandes, Shannon Kelly, Brian Custer, Ester C. Sabino, Carla Luana Dinardo, for the Recipient Epidemiology and Donor Evaluation Study III (REDSIII Study)

**Affiliations:** 1 Centro de Estudos em Células-Tronco, Terapia Celular e Genética Toxicológica (CeTroGen), Programa de Pós-graduação em Saúde e Desenvolvimento na Região Centro-Oeste, Faculdade de Medicina (FAMED), Universidade Federal de Mato Grosso do Sul (UFMS), Campo Grande, Mato Grosso do Sul, Brazil; 2 Institute of Tropical Medicine, Faculdade de Medicina Universidade de São Paulo, São Paulo, São Paulo, Brazil; 3 Universidade Federal de Minas Gerais, Belo Horizonte, Mina Gerais, Brazil; 4 Fundação HEMOMINAS, Belo Horizonte, Minas Gerais, Brazil; 5 Fundação HEMOPE, Recife, Pernambuco, Brazil; 6 Universidade de Pernambuco, Recife, Pernambuco, Brazil; 7 HEMORIO, Rio de Janeiro, Rio de Janeiro, Brazil; 8 Central Laboratory, Hospital das Clínicas da Faculdade de Medicina Universidade de São Paulo, São Paulo, São Paulo, Brazil; 9 Serviço de Pneumologia, Hospital das Clínicas da Faculdade de Medicina Universidade de São Paulo, São Paulo, São Paulo, Brazil; 10 Vitalant Research Institute, San Francisco, California, United States of America; 11 UCSF Benioff Children’s Hospital Oakland, Oakland, California, United States of America; 12 Fundação Pró-Sangue Hemocentro de São Paulo, São Paulo, São Paulo, Brazil; Ankara University: Ankara Universitesi, TÜRKIYE

## Abstract

**Background:**

Smoking is associated with increased morbidity among individuals with sickle cell disease (SCD). While genetic factors influencing smoking behavior have been identified in other populations, they have not been studied in individuals with SCD. This study aimed to assess the impact of smoking on clinical and laboratory parameters in a large SCD cohort and to identify genetic variants potentially associated with smoking behavior in this population.

**Methods:**

The Recipient Epidemiology and Donor Evaluation Study-III (REDS-III) Brazil SCD cohort was established across six Brazilian cities to investigate clinical outcomes in individuals with SCD. Adult participants were interviewed and asked whether they had ever smoked more than 100 cigarettes in their lifetime. Those who responded “yes” were classified as ever smokers, while those who responded “no” were classified as never smokers. Clinical and laboratory data were compared between the two groups. All participants were genotyped using a customized array, and a genome-wide association study (GWAS) was conducted to identify single nucleotide variants (SNVs) associated with smoking status.

**Results:**

Of the 1,231 adults with available smoking data, 332 (27%) were classified as ‘ever smokers’ and 899 (73%) as ‘never smokers’. ‘Ever smoker’ status was associated with male sex, age ≥ 40 years, lower educational attainment, and hemoglobin levels above the 75th percentile. In the GWAS, no SNVs reached genome-wide significance (p < 5 × 10 ⁻ ⁸). However, several SNVs demonstrated nominal significance (p < 1 × 10 ⁻ ⁷), including rs11087854 and a variant at position 1059991 on chromosome 20 within the gene AL110114.1, as well as rs701023 in the NCOR2 gene, suggesting a potential association with smoking behavior.

**Conclusion:**

Biological sex, age, and educational level are associated with smoking status among adults with SCD, and tobacco use appears to correlate with elevated hemoglobin levels in this population. Although no genome-wide significant associations were identified, our findings highlight potential genetic loci—particularly within AL110114.1 and NCOR2—that warrant further investigation in relation to smoking behavior in individuals with SCD.

## Introduction

Tobacco use is a leading cause of preventable death worldwide, contributing to approximately 480,000 deaths annually, primarily due to lung cancer, chronic obstructive pulmonary disease (COPD), and ischemic heart disease [1]. Smoking prevalence remains high in many low- and middle-income countries, and in Latin America, it represents a major risk factor for both mortality and disability [1–3]. On average, smokers have a life expectancy that is 10 years shorter than that of non-smokers. However, cessation before the age of 40 significantly reduces the risk of death from smoking-related complications [4].

Smoking is a multifactorial behavior influenced by genetic susceptibility, psychological factors, socioeconomic conditions, biological mechanisms, and environmental influences [5–8]. An individual’s genetic background plays a significant role [9]. Multiple studies have identified genetic loci associated with smoking initiation, the number of cigarettes smoked per day, and smoking cessation. These include genes involved in nicotine response and the regulation of neurotransmitter pathways activated by nicotine [9–11]. Among these, the cluster of nicotinic acetylcholine receptor genes on chromosome 15 has shown the most consistent associations with various smoking-related phenotypes in genome-wide association studies (GWAS) [12,13]. Genetic factors are known to play a significant role in both the maintenance of smoking and the likelihood of successful cessation, supporting the potential for identifying individual genetic susceptibility to nicotine dependence and informing personalized approaches to smoking cessation therapy [14–16].

In addition to the well-documented chronic inflammation of the airways caused by long-term tobacco use, cigarette smoking also affects systemic immunity by either attenuating protective immune responses or amplifying pathogenic immune processes [17]. Tobacco use is associated with elevated levels of pro-inflammatory cytokines, vascular inflammation, and endothelial dysfunction. It also promotes hemolysis and alters red blood cell (RBC) membrane composition [18–22].

In individuals with SCD, the vascular inflammation and upregulation of endothelial adhesion molecules triggered by active or second-hand smoking may exacerbate vaso-occlusion [23]. Emerging evidence indicates that tobacco smoke contributes significantly to increased morbidity in SCD. Smoking has been identified as a common and important risk factor for vaso-occlusive pain episodes (VOEs), acute chest syndrome (ACS), and lower airway obstruction in children with SCD [23–26]. In adults, at least one study has linked active smoking to a higher incidence of ACS [23]. While the adverse effects of second-hand smoke have been more extensively studied, data on the impact of active smoking in the broader SCD population remain limited.

The primary objective of this study was to assess the impact of smoking on SCD-related clinical complications and to perform a genome-wide association study (GWAS) to identify genetic variants associated with smoking status in adults with SCD.

## Methods

### Recipient epidemiology and donor evaluation study III (REDSIII) SCD cohort

The REDS-III Brazil SCD cohort enrolled individuals with sickle cell disease across six Brazilian sites: Recife (HEMOPE), Rio de Janeiro (HEMORIO), Belo Horizonte, Montes Claros, and Juiz de Fora (Fundação HEMOMINAS), and São Paulo (Child Institute at Hospital das Clínicas, Faculdade de Medicina da Universidade de São Paulo – HCFMUSP). Details of the REDS-III cohort have been previously published [27]. Participant enrollment occurred between November 1, 2013, and March 31, 2015. Medical records were reviewed through August 1, 2018. Following data collection, the study team did not have access to any information that could identify individual participants.

In total, the cohort included 2,793 participants, comprising 1,560 (55.9%) children (<18 years) and 1,233 (44.1%) adults. The distribution of genotypes was as follows: HbSS (70.7%), HbSC (23%), Sβ⁰-thalassemia (3%), Sβ⁺-thalassemia (2.9%), and other genotypes (0.4%). Females accounted for 53% of the cohort. This analysis was restricted to adults (≥18 years), as only they were asked about smoking history.

Adult participants were interviewed about smoking using questions adapted from validated instruments developed by the U.S. Centers for Disease Control and Prevention’s National Health Interview Survey. These same questions have also been employed in large-scale Brazilian studies, including the Pesquisa Nacional de Saúde [28,29]. All interviews were conducted by trained staff in a private setting to ensure participant confidentiality.

The interview included the following questions: “Have you ever smoked more than 100 cigarettes in your lifetime?” and “How many cigarettes have you smoked in the past 30 days?” Participants who answered “yes” to the first question were classified as ‘*ever smokers’*, while those who answered “no” were classified as ‘*never smokers’*. Participants reporting any cigarette use in the past 30 days were categorized as ‘*active smokers’*. Those who were ‘*ever smokers’* but had not smoked in the last 30 days were classified as ‘*ex-smokers’*. Data on second-hand smoke exposure were not collected. All clinical and laboratory data were obtained from patients’ medical records. SCD-related clinical complications were defined according to standardized definitions of SCD phenotypic manifestations [30].

The study protocol was reviewed and approved by the Brazilian National Ethical Committee for Research (CAEE 02790812.0.1001.0065), the local Ethics Committees at each participating center, and the Institutional Review Board of the Vitalant Research Institute (VRI) in San Francisco, CA. Written informed consent was obtained from all participants or, in the case of minors (<18 years), from their legal guardians, allowing inclusion in the REDS-III Brazil SCD cohort and permitting the use of their DNA and data for genome-wide association studies (GWAS).

### Statistical analysis

Qualitative variables are presented as frequencies, and quantitative variables as mean ± standard deviation. Categorical clinical variables were compared between ‘ever smoker’ and ‘never smoker’ groups using the Chi-square test. Continuous variables were analyzed with the independent t-test for normally distributed data or the Mann-Whitney test for non-normally distributed data. Multivariable analysis was performed using logistic regression. A p-value < 0.05 was considered statistically significant. All analyses were conducted using SPSS version 20.0.

### Genotyping

Blood from all participants was collected in EDTA tubes, separated into cellular and plasma components and aliquoted for storage. DNA was extracted from cellular aliquots using alcohol precipitation and quantified using real-time polymerase chain reaction (PCR). DNA was normalized to 10 ng/μl. with a minimum volume of 60 μl.

Genotyping was performed using a customized Affymetrix (Santa Clara, CA) transfusion medicine array that contains approximately 879,000 SNP and copy number polymorphism markers with high genome-wide imputation coverage for African-American (97.5%), Hispanic (96.1%), East Asian (94.6%), and white (96.1%) genomes at a minor allele frequency of 5% [31]. Genotype calls were generated using Axiom Analysis Suite software (Affymetrix), human leukocyte antigen alleles were called with Axiom HLA Analysis software (Affymetrix), and copy number polymorphisms were called with Axiom CNV Summary Tools, with further analysis being performed with PennCNV (http://penncnv.openbioinformatics.org/en/latest/).

Samples with dish quality control (DQC) values less than 0.85 or call rates < 97% were excluded. Plates with plate quality control metrics < 95% or average call rates < 98.5% were also excluded. For SNP quality control, SNPolisher was used, and SNPs in the recommended categories (PolyHighRes, MonoHighRes, NoMinorHom, and Hemizygous) were retained. Samples with inconsistent sex definitions between genotyping and the study database were excluded. PLINK [32] was used to calculate identity-by-descent, and the sample with a lower call rate from pairs of samples with PI_HAT > 0,4 or IBS > 0,9 was excluded. SNPs with call rates < 97% or significant deviation from Hardy-Weinberg equilibrium (P ≤ x 10^−4^) were excluded. Phasing was then conducted with SHAPEIT [33] and imputation with IMPUTE2 [34], with haplotypes derived from the 1000 Genomes Project Phase 3 [35] as reference data.

### Genome-wide association study (GWAS)

Population substructure was determined by generating principal components using PLINK [32] software on linkage disequilibrium pruned autosomal SNPs with unrelated participants only. The top 10 principal components (PCs) were included in the association analyses to adjust for population substructure.

Genome-wide association analyses were conducted using R package GMMAT [36] software with a logistic mixed model, adjusted for age as a categorical variable (<or ≥ 40 years), sex and hemoglobin levels (<or ≥ 75 percentile) as covariates, as well as cryptic kinship relatedness.

The online tool HaploReg v4.1 [37] was used to explore the genes nearest to the index SNPs. LocusZoom [38] was used to generate a QQ-plot, a Manhattan plot and LocusZoom plots, with 1000 Genomes Project Phase 3 LD estimation. A genome-wide P-value threshold of 5x10^-8^ was used to define statistical significance in the GWA analysis [39]. Only variants with minor frequency alleles > 0.05 were considered. An extensive literature search was conducted to investigate the functions of the significant SNPs.

## Results

### Study population

The cohort included 1,232 adult participants, with smoking data available for 1,231 individuals. Among these, 332 (27%) were classified as ‘*ever smokers*’ and 899 (73%) as ‘never smokers’. Of the *‘ever smokers*’, 80 (24.2%) reported smoking at least one cigarette in the past 30 days and were classified as ‘*active smokers*’. The primary clinical and laboratory analyses compared *‘ever smokers*’ and ‘*never smokers*’. Secondary analyses comparing ‘*active smokers*’, ‘*ex-smokers*’, and ‘*never smokers*’ were also performed. These results are provided in the Supplementary Material (Tables S1 and S2) due to smaller sample sizes in the *‘active’* and ‘*ex-smoker*’ groups. To ensure statistical robustness, only variables with a minimum count of 5 observations per group were included in Supplementary Material analyses.

### Association between clinical and laboratory variables and smoking status

Tables 1 and 2 present comparisons between *‘ever smokers*’ and ‘*never smokers*’ with respect to demographic, clinical, and laboratory variables. In univariate analyses, the ‘*ever smoker*’ group had a higher proportion of males (p < 0.001), was older (p < 0.001), and had lower educational attainment (p < 0.001). An association between SCD genotype and smoking status was observed (p < 0.008). Additionally, hemoglobin levels, leukocyte and platelet counts, as well as indirect bilirubin concentrations, differed significantly between ‘*ever smokers*’ (9.36 ± 2.2 g/dL; 9,605 ± 3,811/µL; 337,042 ± 150,852/µL; 0.85 ± 0.88 mg/dL, respectively) and never smokers (9.03 ± 1.9 g/dL; 10,221 ± 4,016/µL; 371,094 ± 149,176/µL; 1.00 ± 1.54 mg/dL, respectively).

**Table 1:** Comparison of demographics between ‘Never Smoker’ and ‘Ever Smoker’ groups within the REDS-III Brazil SCD Cohort.

	Never Smoker	Ever Smoker	p value
	(n = 899)	(n = 332)	
**Biological Sex (male)**	349 (38.8%)	164 (49.4%)	<0.001
**Age**	30.55 ± 10.49	36.27 ± 12.66	<0.001
**Self-declared race** ^**a**^			
White	71 (7.9%)	22 (6.6%)	0.412
Black	318 (35.4%)	112 (33.7%)	
Mixed	476 (53%)	185 (55.7%)	
Indian	2 (0.2%)	3 (0.9%)	
Other	31 (3.5%)	10 (3.1%)	
**SCD type**			0.008
SS	679 (75.5%)	224 (67.5%)	
SC	159 (17.7%)	87 (26.2%)	
Sβ0	33 (3.7%)	8 (2.4%)	
Sβ+	13 (1.4%)	4 (1.2%)	
Sβ+ severe	12 (1.3%)	5 (1.5%)	
SD	4 (0.9%)	3 (0.9%)	
**School Level** ^**b**^			
Never attended school	6 (0.7%)	4 (1.2%)	<0.001
1st-5th year	158 (17.6%)	99 (29.8%)	
6st-9th year	118 (13.1%)	65 (19.6%)	
Highschool	432 (48.1%)	129 (38.9%)	
Learnt to write while adult	6 (0.7%)	2 (0.6%)	
Technical Course	65 (7.2%)	8 (2.4%)	
College	111 (12.3%)	25 (7.5%)	
Master degree	3 (0.3%)	0	

a)Race categories are based on self-declaration and correspond to IBGE census classifications by skin color: White (Branca), Black (Preta), Mixed race (Parda), Indigenous (Indígena), and Other (Yellow/Asian descent). b) School level are based on IBGE census classifications: Never attended school (Nunca frequentou), 1st-5th year (Regular do ensino fundamental), 6st-9th year (Regular do ensino fundamental), Highschool (Regular do ensino médio), Learnt to write while adult (alfabetização de jovens e adultos), Technical Course (Especialização de nível superior), College (Superior de graduação) e Master degree (Mestrado)

**Table 2:** Comparison of clinical and laboratory outcomes between ‘Never Smoker’ and ‘Ever Smoker’ groups within the REDS-III Brazil SCD Cohort.

	Never Smoker	Ever Smoker	p value
	(n = 899)	(n = 332)	
**Chronic Obstructive Pulmonary Disease (COPD) - Yes (n/%)**	7 (21.9%)	3 (13%)	0.494
**Restrictive Pulmonary Disease – Yes (n/%)**	9 (28.1%)	5 (21.7%)	0.592
**Inhaled Bronchodilator – Yes (n/%)**	9 (1%)	5 (1.5%)	0.458
**Home use of oxygen - Yes (n/%)**	2 (0.2%)	1 (0.3%)	0.804
**Hospitalizations in the last 12 months – Yes (n/%)**	250 (27.8%)	109 (32.8%)	0.085
**Abnormal transcranial doppler – Yes (n/%)**	41 (16.5%)	9 (14.3%)	0.665
**Pulmonary Hypertension – Yes (n/%)**	69 (15.8%)	37 (21.6%)	0.085
**Lifetime number of transfusions**			
1–5	231 (32.5%)	86 (34%)	0.865
6–10	140 (19.7%)	50 (19.8%)	
11–20	115 (16.2%)	43 (17%)	
21–40	95 (13.4%)	34 (13.4%)	
41–60	32 (4.5%)	13 (5.1%)	
61–80	17 (2.4%)	2 (0.8%)	
81–100	18 (2.5%)	6 (2.4%)	
> 100	63 (8.9%)	19 (7.5%)	
**Red Blood Cell antibodies – Yes (n/%)**	68 (13.3%)	26 (12.4%)	0.747
**Hemoglobin**	9.03 ± 1.89	9.36 ± 2.2	0.016
**Leukocyte count**	10,221 ± 4,016	9,605 ± 3,811	0.025
**Platelet count**	371,094 ± 149,176	337,042 ± 150,852	0.001
**Total Bilirrubin**	1.35 ± 0.47	1.42 ± 0.45	0.029
**Indirect Bilirrubin**	1 ± 1.154	0.85 ± 0.88	0.038
**Ferritin**	932.27 ± 1,839	963 ± 1,616	0.784
**Hydroxyurea – Yes (n/%)**	103 (31%)	323 (36%)	0.108
**Pain Crisis – Yes (n/%)**	827 (92.8%)	304 (91.6%)	0.461
**Neuropathic Pain – Yes (n/%)**	15 (1.7%)	4 (1.2%)	0.554
**Retinopathy – Yes (n/%)**	80 (14.7%)	38 (18%)	0.265
**Pulmonary Embolism – Yes (n/%)**	12 (1.3%)	3 (0.9%)	0.538
**Chronic Heart Failure – Yes (n/%)**	57 (6.4%)	26 (0.08%)	0.322
**Stroke – Yes (n/%)**	84 (9.4%)	30 (9%)	0.863
**Hemorrhagic Stroke – Yes (n/%)**	7 (0.8%)	2 (0.6%)	0.747
**Venous Thromboembolism – Yes (n/%)**	26 (2.9%)	7 (2.1%)	0.451
**Leg Ulcers – Yes (n/%)**	155 (17.3%)	64 (19.3%)	0.413
**Acute Chest Syndrome – Yes (n/%)**	192 (60.4%)	527 (61%)	0.847
**Hospital Admissions due to Pain Crisis (mean ± SD)**	11.4 ± 30.5	9.85 ± 18.85	0.365
**Hospital Admissions due to Acute Chest Syndrome (mean ± SD)**	3.52 ± 3.8	3.9 ± 4.28	0.310

No significant differences were observed between groups for the following clinical outcomes: vaso-occlusive pain crisis, pulmonary embolism, ischemic stroke, hemorrhagic stroke, venous thromboembolism, and hospitalizations within the past 12 months. Although the prevalence of pulmonary hypertension was higher among ‘*ever smokers*’ (21.6%) compared to *‘never smoker*s’ (15.8%), this difference did not reach statistical significance (p = 0.085), potentially due to limited sample size.

In the multivariable analysis (Table 3), male sex, age ≥ 40 years, lower educational level, and hemoglobin levels above the 75th percentile were independently associated with ‘*ever smoker’* status. When ‘ever smokers’ were further stratified into ‘active smokers’ and ‘ex-smokers’, and compared with ‘never smokers’, several variables differed between the groups, including sex, age, educational level, hemoglobin concentration, platelet count, and indirect bilirubin levels. However, in the multivariable model, only educational level (p = 0.016) and sex (p < 0.001) remained statistically significant.

**Table 3:** Multivariable model of the comparison between ‘Never Smoker’ and ‘Ever Smoker’ groups.

Variables	OR (IC 95% OR)	*p* value
**Intercept**	0.118 (0.070; 0.192)	<0.001
**Biological Sex**		
Female	–	–
Male	1.741 (1.298; 2.339)	<0.001
**Age**		
< 40 years	–	–
40 years or more	2.950 (2.155; 4.042)	<0.001
**School level**		
Low	2.163 (1.261; 3.821)	0.006
Medium	1.309 (0.802; 2.220)	0.297
High	–	–
**Hemoglobin**		
< P75%	–	–
≥ P75%	1.571 (1.144; 2.150)	0.005

School Level: -Low (never attended school to 5^th^ year), – Medium (6^th^ – 9^th^ year, highschool and technical course), – High (college and master degree).

Although hemoglobin level did not reach statistical significance in the multivariable model, this may reflect the smaller sample size of the ‘ex-smoker’ group. Nonetheless, a trend was observed, with mean hemoglobin levels being highest among active smokers (9.49 ± 2.27 g/dL), followed by ex-smokers (9.32 ± 2.19 g/dL), and lowest among never smokers (9.03 ± 1.89 g/dL). Detailed comparisons among ‘active smokers’, ‘ex-smokers’, and ‘never smokers’ are provided in Supplementary Tables 1 and 2.

### Genome-wide association analysis

**Fig 1:**
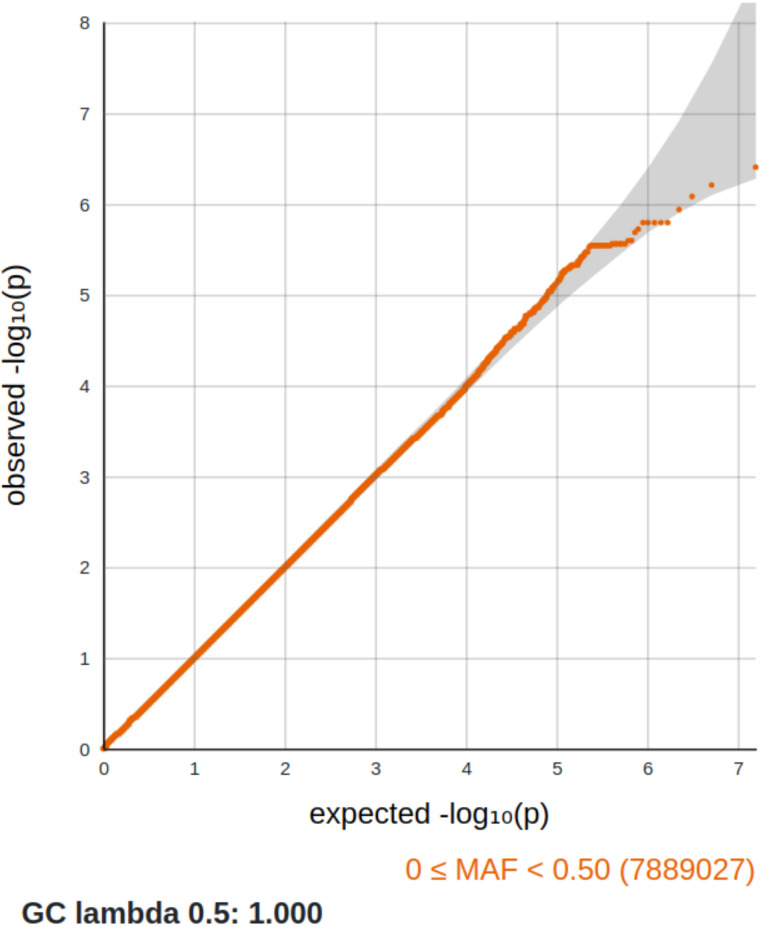
Q-Q plot: Q-Q plot of the observed (y axis) versus expected (x axis) -log(P-values). The graph suggests that there were no genomic inflation or other significant confounding factors.

The GWAS included 305 ‘*ever smokers’* and 837 ‘*never smokers’*. Not all participants were included in this analysis due to missing genetic data or failure to meet quality control criteria. The analysis showed no evidence of p-value inflation (λ_GC_ = 1), and the Q-Q plot (Fig 1) indicated no signs of genomic inflation or other major confounding factors.

**Fig 2:**
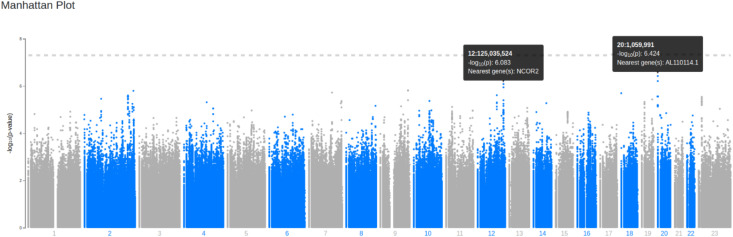
Manhattan plot: Manhattan plot of the GWAS. The Manhattan plot shows the genome wide -log 10 P-values plotted against the position on each chromosome showing the association of SNPs with ever smoker status in the REDS-III Brazil SCD cohort study. No SNVs reached genome-wide significance (*p* < 5x10^-8^). Top 3 SNVs are in gene AL110114.1 and NCOR2.

**Fig 3:**
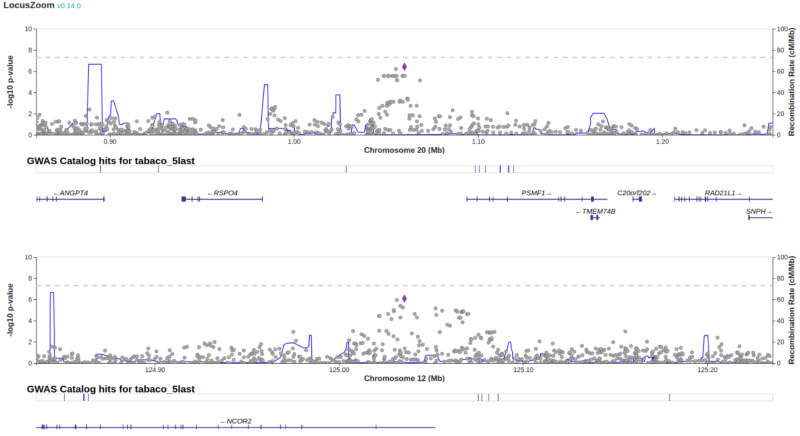
Regional plot: Regional plot on chromosomes 22 and 12. Axis y represents the negative of the logarithm of P-values for the association of the SNVs with ever smoker status. SNVs are represented as dots. Axis x shows the relative positions of the SNVs in this region of the chromosome, representing the recombination rate in centimorgans (cM) per megabase (Mb). SNVs in position 1059991 in chromosome 20 and rs701023 in chromosome 12 are represented by a purple diamond.

The GWAS results are summarized in Fig 2, which displays the distribution of p-values for the association between SNPs and ‘ever smoker’ status. No SNPs reached genome-wide significance (p < 5 × 10 ⁻ ⁸). However, a targeted analysis of previously reported SNPs associated with smoking behavior was conducted using a less stringent significance threshold. Three single nucleotide variants (SNVs) showed nominal significance (p < 1 × 10 ⁻ ⁷): rs11087854 and a variant at position 1059991 on chromosome 20, both located within the gene AL110114.1, and rs701023 within the Nuclear Receptor Corepressor 2 (NCOR2) gene (Figs 2 and 3). These variants suggest a potential association with ‘ever smoker’ status and are summarized in Table 4.

**Table 4:** Single nucleotide variations (SNVs) with suggestive association with ever smoker status among SCD individuals in GWAS.

Chromosome	SNP or Position	Locus	*p*-value
20	1059991	AL110114.1	3.8 x 10^−7^
20	rs11087854	AL110114.1	6.1 x 10^−7^
12	rs701023	NCOR2	8.3 x 10^−7^

## Discussion

This study assessed the impact of smoking on the clinical and laboratory profiles of individuals with SCD, and explored genetic variants potentially associated with smoking status in this population. “Ever smoking” status was found to be more prevalent among older individuals, males, and those with lower educational attainment. Furthermore, smoking was associated with elevated hemoglobin levels. A GWAS was conducted to identify genetic determinants of smoking status, revealing a potential association between smoking and variants in the NCOR2 gene.

To our knowledge, this is the first study to evaluate tobacco smoking in a large cohort of individuals with SCD. Our findings align with previous studies on the demographics of tobacco use in the general population [40]. A higher prevalence of smoking among males has been consistently reported in large international studies conducted across more than 100 countries, including Brazil, where in 2015, daily smoking prevalence was 12.6% among men and 8.2% among women [40,41]. An association between SCD genotype and smoking status was observed. However, this relationship is probably caused by ancestry and, consequently, socioeconomic status. Consistent with our results, prior research has also shown that smoking is more common among individuals with lower educational attainment and older age [41,42]. These findings suggest that well-established demographic predictors of tobacco use in the general population are similarly applicable to individuals with SCD. Such insights may help inform and tailor anti-smoking education and intervention campaigns specifically for the SCD population.

Cigarette smoking is known to increase hemoglobin concentration due to exposure to carbon monoxide, which binds to hemoglobin and reduces its oxygen-carrying capacity [43,44]. As a compensatory response, higher hemoglobin levels are required to maintain adequate oxygen delivery. This association has been demonstrated in large population-based cohorts, which have also reported a positive correlation between the number of cigarettes smoked per day and hemoglobin levels [45,46]. In line with these findings, our study shows that individuals with SCD who reported ever smoking had significantly higher hemoglobin levels compared to never-smokers. This suggests that the physiological mechanisms responsible for hemoglobin upregulation in response to smoking are preserved in SCD patients, despite their underlying chronic hemolysis and baseline anemia.

Our results identified a potential association between the genomic regions near AL110114.1 and NCOR2 and ‘ever-smoker’ status. Previous genetic studies have reported various loci associated with different smoking-related phenotypes [10]. The largest meta-analysis to date was conducted by the Tobacco and Genetics (TAG) Consortium in collaboration with ENGAGE and the Oxford–GlaxoSmithKline (GSK) consortia. That analysis identified loci associated with specific smoking behaviors, including CHRNA3 and EGLN2 for the number of cigarettes smoked per day, BDNF for smoking initiation, and DBH for smoking cessation [11]. While the loci identified in our study differ, this may be attributed to differences in the phenotypes assessed and the ancestral backgrounds of the study populations. The TAG Consortium focused primarily on individuals of European ancestry, whereas the Brazilian SCD population included in our study is predominantly of mixed ancestry [27,47].

AL110114.1, a poorly characterized gene, is located near the two top-associated variants identified in our analysis. Nuclear receptor corepressors (NCORs) are broadly expressed in the mammalian brain and function as transcriptional regulators by binding to nuclear hormone receptors. They modulate gene expression in response to various stimuli, including hormones, metabolites, xenobiotics, and drugs [48–50]. Experimental studies in animal models have implicated NCORs in several neurological and behavioral processes, such as anxiety, sexual behavior, learning, and memory [48]. In humans, NCOR2 has been associated with cocaine dependence in European-Americans, although this association did not replicate in African-American populations [51]. A potential link between NCOR and smoking has also been suggested in a study examining transcriptomic changes in the lungs of cigarette smoke-exposed mice [48]. These findings support a possible role of NCOR2 in the biological response to tobacco exposure, warranting further investigation in the context of sickle cell disease.

NCOR2 is a corepressor involved in transcriptional regulation and plays a significant role in modulating gene expression in the brain [52]. It is part of the Macrophage Migration Inhibitory Factor (MIF)-mediated glucocorticoid regulation pathway [53]. Glucocorticoid receptor-regulated enhancers play a central role in the gene regulatory networks underlying drug addiction [54].

Finally, the prevalence of pulmonary hypertension was over 30% higher in the ‘ever-smoker’ group compared to ‘never-smokers’. While this finding is biologically plausible and clinically relevant, its interpretation is limited by the sample size and the substantial amount of missing echocardiographic data within the cohort, which constrained the robustness of the analysis.

This study has some limitations. The number of individuals classified as ‘ever smokers’ was relatively small compared to large-scale studies of smoking in the general population. As a result, assessing the impact of tobacco use on clinical outcomes with low prevalence was challenging. Nonetheless, this remains the largest study to date examining smoking behavior in individuals with sickle cell disease (SCD), underscoring the relevance of our findings for this specific patient population. The classification of “ever smokers” based on having smoked more than 100 cigarettes in a lifetime is broad and may not fully capture the variability in smoking exposure. The smoking burden—typically quantified as pack-years—was not captured, as the original questionnaire did not include information on the number of cigarettes smoked or the duration of smoking. This limitation prevented a more detailed assessment of dose-dependent effects of tobacco use. We plan to validate our findings by reproducing the results in an independent cohort. An admixture mapping analysis is being planned to investigate ancestry-specific signals.

In conclusion, age, educational level, and sex were significantly associated with smoking status among individuals with SCD, consistent with findings from studies in the general population. Additionally, smoking was associated with higher hemoglobin levels in this patient group. Our genetic analysis suggests a potential association between smoking status and variants in the regions of AL110114.1 and NCOR2, indicating a possible genetic contribution to smoking behavior in SCD patients. These findings provide a foundation for further investigation into the clinical and genetic factors influencing tobacco use in this population.

## Supporting information

S1 Table Univariate comparison between ‘Never Smokers’, ‘Ex-Smokers’ and ‘Active Smokers’ groups in terms of demographic features. (DOCX)

S2 Table Univariate comparison between Never Smokers’, ‘Ex-Smokers’ and ‘Active Smokers’ groups in terms of clinical and laboratorial features. (DOCX)
