# Supplementary material for: Clinical, laboratory and genetic factors associated with smoking in a Brazilian Sickle Cell Disease (SCD) cohort
Source: PLoS One. 2025 Sep 26;20(9):e0332305. doi: 10.1371/journal.pone.0332305 (PMC12469115; doi:10.1371/journal.pone.0332305)
Supplement: S1 Table — (DOCX) [file pone.0332305.s001.docx]

**Supplementary Material**

**S1 Table** Univariate comparison between ‘Never Smokers’, ‘Ex-Smokers’ and ‘Active Smokers’ groups in terms of demographic features

|  | **Ex-smokers** | **Active Smokers** | **Never Smoker** | **p value** |
| --- | --- | --- | --- | --- |
|  | (n=251) | (n=80) | (n=899) |  |
|  |  |  |  |  |
| **Gender (male)** | 112 (44.6%) | 51 (63.75%) | 349 (38.8%) | <0.001 |
|  |  |  |  |  |
| **Age** | 37.6 ± 12.3 | 32.33 ± 12.83 | 30.55 ± 10.49 | <0.001 |
|  |  |  |  |  |
| **Self-declared race** |  |  |  |  |
| White | 13 (5.8%) | 9 (11.25%) | 71 (7.9%) | 0.475 |
| Black | 85 (33.9%) | 27 (33.75%) | 318 (35.4%) |  |
| Mixed | 143 (57%) | 41 (51.25%) | 476 (53%) |  |
| Indian | 2 (0.8%) | 1 (1.25%) | 2 (0.2%) |  |
| Other | 8 (3.2%) | 2 (2.5%) | 31 (3.5%) |  |
|  |  |  |  |  |
| **SCD type** |  |  |  | 0.06 |
| SS | 169 (67.3%) | 54 (67.5%) | 679 (67.5%) |  |
| SC | 68 (27.1%) | 19 (23.7%) | 159 (26.2%) |  |
| Sβ0 | 6 (2.4%) | 2 (2.5%) | 33 (3.7%) |  |
| SD | 4 (1.6%) | 0 (0%) | 4 (0.9%) |  |
| Sβ+ severe | 3 (1.2%) | 2 (2.5%) | 12 (1.3%) |  |
| Sβ+ | 1 (0.4%) | 3 (3.8%) | 13 (1.4%) |  |
|  |  |  |  |  |
| **School Level** |  |  |  |  |
| Never attended school | 3 (1.2%) | 1 (1.25%) | 6 (0.7%) | <0.001 |
| 1st-5th year | 76 (30.3%) | 23 (28.75%) | 158 (17.6%) |  |
| 6th-9th year | 41 (16.3%) | 23 (28.75%) | 118 (13.1%) |  |
| High School | 102 (40.6%) | 27 (33.75%) | 432 (48.1%) |  |
| Learnt to write while adult | 2 (0.8%) | 0 | 6 (0.7%) |  |
| Technical Course | 6 (2.4%) | 2 (2.5%) | 65 (7.2%) |  |
| College | 21 (8.4%) | 4 (5%) | 111 (12.3%) |  |
| Master degree | 0 | 0 | 3 (0.3%) |  |

1. Race categories are based on self-declaration and correspond to IBGE census classifications by skin color: White (Branca), Black (Preta), Mixed race (Parda), Indigenous (Indígena), and Other (Yellow/Asian descent). b) School level are based on IBGE census classifications: Never attended school (Nunca frequentou), 1st-5th year (Regular do ensino fundamental), 6st-9th year (Regular do ensino fundamental), Highschool (Regular do ensino médio), Learnt to write while adult (alfabetização de jovens e adultos), Technical Course (Especialização de nível superior), College (Superior de graduação) e Master degree (Mestrado)

In multivariable analysis, gender and age reached statistical significance
