# Supplementary material for: Clinical, laboratory and genetic factors associated with smoking in a Brazilian Sickle Cell Disease (SCD) cohort
Source: PLoS One. 2025 Sep 26;20(9):e0332305. doi: 10.1371/journal.pone.0332305 (PMC12469115; doi:10.1371/journal.pone.0332305)
Supplement: S2 Table — (DOCX) [file pone.0332305.s002.docx]

**Supplementary Material**

**S2 Table** Univariate comparison between Never Smokers’, ‘Ex-Smokers’ and ‘Active Smokers’ groups in terms of clinical and laboratorial features

|  | **Ex-smokers** | **Active Smokers** | **Never Smoker** | **p value** |
| --- | --- | --- | --- | --- |
|  | (n=251) | (n=80) | (n=899) |  |
| **Chronic Obstructive Pulmonary Disease (COPD) - Yes (n/%)** | 2 (11.1%) | 1 (20%) | 7 (21.9%) | 0.661 |
|  |  |  |  |  |
| **Restrictive Pulmonary Disease - Yes (n/%)** | 3 (16.7%) | 2 (40%) | 9 (28.1%) | 0.507 |
|  |  |  |  |  |
| **Hospitalizations in the last 12 months - Yes (n/%)** | 77 (30.7%) | 31 (38.75%) | 250 (27.8%) | 0.101 |
|  |  |  |  |  |
| **Abnormal transcranial doppler - Yes (n/%)** | 7 (15.2%) | 2 (12.5%) | 41 (16.5%) | 0.881 |
|  |  |  |  |  |
| **Pulmonary Hypertension - Yes (n/%)** | 29 (20.6%) | 8 (26.7%) | 69 (15.8%) | 0.162 |
|  |  |  |  |  |
| **Lifetime number of transfusions** |  |  |  |  |
| 1 to 5 | 69 (36%) | 17 (28.3%) | 231 (32.5%) | 0.31 |
| 6 to 10 | 36 (18.75%) | 14 (23.3%) | 140 (19.7%) |  |
| 11 to 20 | 34 (17.7%) | 8 (13.3%) | 115 (16.2%) |  |
| 21 to 40 | 22 (11.45%) | 12 (20%) | 95 (13.4%) |  |
| 41 to 60 | 11 (5.7%) | 2 (3.3%) | 32 (4.5%) |  |
| 61 to 80 | 2 (1%) | 0 | 17 (2.4%) |  |
| 81 to 100 | 5 (2.6%) | 1 (1.7%) | 18 (2.5%) |  |
| >100 | 13 (7%) | 6 (10%) | 63 (8.9%) |  |
|  |  |  |  |  |
| **Red Blood Cell antibodies - Yes (n/%)** | 22 (13.8%) | 4 (8.2%) | 68 (13.3%) | 0.556 |
|  |  |  |  |  |
| **Hemoglobin** | 9.32 ± 2.188 | 9.49 ± 2.274 | 9.03 ± 1.89 | 0.055 |
|  |  |  |  |  |
| **Leukocyte count** | 9,520 ± 3,925 | 9,913 ± 3,456 | 10,221 ± 4,016 | 0.072 |
|  |  |  |  |  |
| **Platelet count** | 335,500 ± 151,606 | 343,429 ± 150,041 | 371,094 ± 149,176 | 0.005 |
|  |  |  |  |  |
| **Indirect Bilirubin** | 0.81 ± 0.829 | 1.01 ± 1.046 | 1 ± 1.154 | 0.047 |
|  |  |  |  |  |
| **Hydroxyurea - Yes (n/%)** | 82 (32.7%) | 20 (25%) | 103 (31%) | 0.104 |
|  |  |  |  |  |
| **Pain Crisis - Yes (n/%)** | 229 (91.2%) | 74 (92.5%) | 827 (92.8%) | 0.698 |
|  |  |  |  |  |
| **Neuropathic Pain - Yes (n/%)** | 4 (1.6%) | 0 (0%) | 15 (1.7%) | 0.506 |
|  |  |  |  |  |
| **Retinopathy - Yes (n/%)** | 31 (18.8%) | 7 (15.2%) | 80 (14.7%) | 0.428 |
|  |  |  |  |  |
| **Pulmonary Embolism - Yes (n/%)** | 3 (1.2%) | 0 (0%) | 12 (1.3%) | 0.579 |
|  |  |  |  |  |
| **Chronic Heart Failure - Yes (n/%)** | 23 (9.3%) | 3 (3.9%) | 57 (6.4%) | 0.154 |
|  |  |  |  |  |
| **Stroke - Yes (n/%)** | 24 (9.6%) | 5 (6.25%) | 84 (9.4%) | 0.612 |
|  |  |  |  |  |
| **Hemorrhagic Stroke - Yes (n/%)** | 2 (0.8%) | 0 (0%) | 7 (0.8%) | 0.729 |
|  |  |  |  |  |
| **Venous Thromboembolism - Yes (n/%)** | 7 (2.8%) | 0 (0%) | 26 (2.9%) | 0.305 |
|  |  |  |  |  |
| **Leg Ulcers - Yes (n/%)** | 49 (19.6%) | 15 (18.75%) | 155 (17.3%) | 0.682 |
|  |  |  |  |  |
| **Acute Chest Syndrome - Yes (n/%)** | 144 (60.25%) | 47 (60.3%) | 529 (61.1%) | 0.967 |

In multivariable analysis, no clinical or laboratory variables reached statistical significance.
